# Hygiene of the Face

**Published:** 1896-05

**Authors:** 


### Selections.

### Hygiene of the Face.

To a very large proportion of the human race in civilized countries the face is, under the designation of "the complexion," the subject of considerable and painstaking interest. Even those most exempt from vanity would prefer to have a physiognomy not readily identifiable by a more or less symmetrical crop of pimples; and a congested nose is not regarded as a thing of beauty, even in an omnibus driver. Yet, in spite of this general feeling in favor of a normal complexion, ignorance and carelessness between them wreak havoc, and it is the exception to meet with cheeks that have seen more than twenty summers which do not betray traces of ill-treatment. Apart from indigestion and constipation—two potent factors in the ruin of a naturally healthy complexion—there is a variety of forms of mismanagement which conduce to blotchiness and pimply deformities. Among them must be ranked the practice of washing the face with hot water, a widespread form of self-indulgence in cold weather. The hot water, especially when reinforced by a course of unduly alkaline soap, removes an unduly large proportion of the natural fat of the skin, leaving it with a roughened surface, which is very liable to excoriate or "chap," and requires more frequent washing to keep it clean, owing to its catching the dust. Nothing, probably, does so much to age the skin as the frequently repeated ablutions with hot water, and this may explain why the dainty Frenchwoman prefers to smear off the grime with the corner of a handkerchief steeped in glycerine, knowing by experience that good, honest soap and water is, in the long run, detrimental to the preservation of a healthy skin.—*Charlotte Med. Journal.*

---

It is stated in the *Hospital* that the Medical Society of Berne is endeavoring to prevent the publication of notices of cases of suicide. It has been observed that suicides have been frequently suggested by these means.

